# Role of Insulin Clearance in Insulin Action and Metabolic Diseases

**DOI:** 10.3390/ijms24087156

**Published:** 2023-04-12

**Authors:** Hilda E. Ghadieh, Amalia Gastaldelli, Sonia M. Najjar

**Affiliations:** 1 Department of Biomedical Sciences, Faculty of Medicine and Medical Sciences, University of Balamand, Kalhat, Al Koura, Tripoli P.O. Box 100, Lebanon; 2 Cardiometabolic Risk Unit, Institute of Clinical Physiology-National Research Council, 56124 Pisa, Italy; 3 Department of Biomedical Sciences, Heritage College of Osteopathic Medicine, Ohio University, Athens, OH 45701, USA; 4 Diabetes Institute, Heritage College of Osteopathic Medicine, Ohio University, Athens, OH 45701, USA

The year 2021 marked the centenary of the discovery of insulin. Since its discovery, investigations have focused on how insulin secretion from pancreatic beta cells triggers branching signaling pathways to mediate its physiological actions. Secreted in pulses into the portal circulation, insulin binds to cell surface receptors on hepatocytes through fenestrations of endothelial cells lining the hepatic sinusoid. This triggers its rapid receptor-mediated internalization and targeting for degradation to be cleared from circulation. Insulin degradation constitutes the basic mechanism of hepatic insulin clearance. In recent years, this process has gained traction since it has been found to contribute to determining the physiologic levels of insulin reaching post-hepatic tissues and, subsequently, regulate systemic insulin action. This Special Issue, entitled “Role of Insulin Clearance in Insulin Action and Metabolic Diseases”, focuses on the body of work delineating the molecular mechanisms of altered insulin clearance and its role in the pathogenesis of dysregulated metabolism.

In “The Discovery of Insulin: New Insights on Its 100th Birthday: From Insulin Action and Clearance to Sweet Networks”, Leroux et al. [1] review the evidence that led to the discovery as well as purification of insulin and its cell surface receptors, in addition to describing the phases of insulin action and clearance: from its binding to insulin receptors to endocytosis, its transport within endosomes, and its ultimate degradation. In this review, the authors present a novel concept of disturbed protein–protein interaction networks in a type 2 diabetes (T2D) functional module. In addition to genome-wide association studies, this integrated network identifies several major genes involved in non-canonical pathways in insulin endosomal transport. The review addresses how rare and common variants contribute to this T2D module. It also discusses how hepatic insulin clearance coordinates efforts with insulin secretion to support the co-functionality of the T2D module with islets and how this may be applied to T2D subgrouping as well as network precision medicine.

In the article entitled “The Physiology of Insulin Clearance”, Bergman et al. [2] review how studies by Himsworth first suggested that resistance to insulin action characterized T2D, as well as how this concept was later confirmed by Reaven and colleagues. As such, the 1930s witnessed the identification of the two primary types of diabetes: “juvenile onset” (type 1) and “adult onset” (type 2). Subsequent studies by Porte and others invoked pancreatic beta cell dysfunction as the primary pathogenesis of T2D. This controversy (insulin resistance versus beta cell failure) persisted until it was possible to examine the nature of their relationship, as defined by Bergman and colleagues via the disposition index (DI), denoting the product of insulin secretion x insulin sensitivity. Further studies showed that a lower DI portends the conversion from impaired glucose tolerance into frank T2D.

However, it has recently become clear that, in addition to insulin secretion, factors such as the rate of insulin degradation, primarily by the liver, should be considered as regulators of the insulin response. An important question arose concerning the importance of insulin clearance in the pathogenesis of T2D. Attempts to address this question are reviewed by Bergman et al. [2] and Koh et al. [3]. Bergman et al. [2] report how, decades earlier, Mirsky first proposed that insulin resistance is linked to reduced insulin clearance, but that consideration of the importance of insulin clearance remained moribund for decades. Indeed, it is only recently that the role of insulin clearance has become the focus of research by several groups. Plasma insulin clearance is reduced in people with insulin resistance, and, on average, half of secreted insulin is cleared by the liver on the first pass; however, that fraction may vary from 20% to 77%. The review also discusses how insulin clearance rate is altered by environmental factors (e.g., dietary composition) and varies among ethnic groups (African Americans—low clearance; European Americans—high clearance). The ethnic difference was also observed in children, with African Americans having the lowest clearance. Because diabetes rates are the highest in ethnic groups manifesting low insulin clearance, it has been proposed that lower clearance is likely a pathogenic factor in the development of diabetes. The hypothesis is that low clearance results in a greater escape of insulin from liver degradation, leading to chronic systemic hyperinsulinemia that in turn causes insulin resistance in skeletal muscle and other peripheral tissues, an event that stresses the pancreatic beta cells and causes T2D. Studies on some ethnic groups, including the Pima Native Americans, have supported the concept that reduced insulin clearance portends conversion into frank diabetes independent of other risk factors. In this respect, the authors proposed that a pharmacologic increase in hepatic insulin clearance may be a therapeutic pathway for preventing or treating T2D.

In contrast to Bergman et al., Koh et al. [3] argue, in their article entitled “Insulin Clearance in Obesity and Type 2 Diabetes”, that lower insulin clearance compensates for insulin resistance rather than causes it. They review how insulin is a key regulator of plasma glucose concentration by suppressing hepatic glucose production and stimulating glucose disposal (primarily in skeletal muscle). Whereas insulin action is impaired in individuals with obesity, most obese subjects display a normal plasma glucose concentration, likely owing to elevated insulin levels resulting from a compensatory increase in insulin secretion and a reduction in insulin clearance. Of note, both insulin action and insulin clearance require insulin to bind to receptors on cell surfaces with subsequent insulin–insulin receptor complex internalization and insulin degradation. Insulin receptor expression is reduced in individuals with insulin resistance. Additionally, an increase in insulin—chronically or acutely, such as after ingesting a meal—causes receptor internalization and thereby reduces plasma insulin clearance. Individuals with obesity and T2D have impaired beta cell function and therefore insulin insufficiency. Their plasma insulin clearance is higher than in subjects with obesity but without diabetes, presumably because of beta cell dysfunction and a low tissue insulin load in addition to their impaired intracellular insulin degradation. In summary, the review emphasizes that beta cell function drives plasma insulin concentration directly, by determining the insulin secretion rate, and indirectly, through the dose-dependency of plasma insulin clearance.

The mechanisms of insulin clearance are reviewed by Leissering et al. [4] in the article entitled “Targeting Insulin-Degrading Enzyme in Insulin Clearance”. The review focuses on insulin degrading enzyme (IDE), a zinc metallopeptidase that avidly degrades insulin in vitro, but its role in vivo has been poorly characterized. In this article, the authors present a comprehensive review of the evidence to support or rule out a critical role for IDE in regulating hepatic and renal insulin clearance. They also discuss the effects of pharmacological and genetic manipulations of IDE on insulin and glucose homeostasis in vivo.

In addition to IDE, carcinoembryonic antigen-related cell adhesion molecule 1 (CEACAM1) has been shown to be a key regulator of insulin clearance by increasing the rate of insulin–insulin receptor complex in hepatocytes as well as in proximal tubule cells (pioneered by Najjar and colleagues [5]). In their original research paper entitled “Nicotinamide Mononucleotide Prevents Free Fatty Acid-Induced Reduction in Glucose Tolerance by Decreasing Insulin Clearance”, Nahle et al. [6] assessed the effects of nicotinamide mononucleotide (NMM), a precursor of nicotinamide, on insulin clearance. Their findings suggest that NMM treatment increased plasma levels of the SIRT2 inhibitor nicotinamide, which synergized with fatty acids to induce oxidative stress and in turn downregulate CEACAM1. This synergistic effect increased glucose tolerance acutely, as opposed to chronically causing the hyperinsulinemia-induced amplification of insulin resistance. In addition, they found that, in the absence of fatty acids, NMM caused pancreatic beta cell dysfunction. Thus, conditions of low SIRT2 activity and the elevated production of reactive oxygen species, such as aging, may increase susceptibility to the effect of fatty acids on insulin clearance. Under these conditions, reduced clearance may serve as the primary defect that increases the risk for T2D. This, in part, supports the argument presented by Bergman et al. [2].

Finally, in their original research paper entitled “Altered Insulin Clearance after Gastric Bypass and Sleeve Gastrectomy in the Fasting and Prandial Conditions”, Salehi et al. [7] compared the effects of Roux-en-Y gastric-bypass (GB) and sleeve gastrectomy (SG) on insulin kinetics and the insulin clearance rate (ICR) in fasting and fed states. Three groups of subjects with comparable body mass indices and glucose tolerance were studied: subjects with a history of GB or SG and a control group of age-matched, non-operated, and non-diabetic subjects (CN). These groups were studied at fasting, during a mixed-meal test (MMT), and during an MMT combined with a hyperinsulinemic–hypoglycemic clamp analysis. Both surgeries induced insulin secretion rates (ISRs) in parallel to fasting and postprandial hepatic ICR, and manifested a reduction in excursion in peripheral insulin concentrations. Compared to CN, both surgeries induced a greater extraction rate of exogenous insulin. Collectively, the data demonstrated that GB and SG influence the ICR independently of meal stimulation, and that an increased ICR following bariatric surgery was due to changes in hepatic insulin clearance rather than peripheral insulin sensitivity, which mainly depends on insulin uptake in skeletal muscle, and was similar among the three groups.

This Special Issue highlights the relevance of insulin clearance to systemic insulin action. Whether reduced insulin action is compensated for by reduced insulin clearance in addition to increased insulin secretion, or whether reduced insulin clearance causes the hyperinsulinemia-driven down-regulation of insulin receptors, leading to resistance and hepatic steatosis, remains a matter of debate [8]. Thus far, therapeutics have focused on targeting insulin secretion and action; little effort has been devoted to hepatic insulin clearance. Understanding insulin clearance and identifying its basic mechanisms can provide a different approach with which to lower insulin resistance and ameliorate its associated metabolic diseases, as is proposed by Bergman et al. [2]. This Special Issue provides a platform on which to discuss the design and implementation of tools to pursue this goal.
